# A case report: identifying a novel variant in ELOC(*TCEB1*)-mutant renal cell carcinoma

**DOI:** 10.3389/fonc.2025.1661834

**Published:** 2025-11-11

**Authors:** Hai-Peng Cheng, Na-Mei Li, Ke-Xin Zeng, Peng Zhou, Xiao-Hong Li

**Affiliations:** 1 Department of Pathology, The Second Xiangya Hospital, Central South University, Changsha, China; 2 Hunan Clinical Medical Research Center for Cancer Pathogenic Genes Testing and Diagnosis, Changsha, China; 3 Geneplus-Beijing, Beijing, China

**Keywords:** renal cell carcinoma, ELOC(TCEB1) gene, novel variant, case report, next generation sequencing (NGS)

## Abstract

*ELOC*(*TCEB1*)-mutant renal cell carcinoma [*ELOC*(*TCEB1*)-RCC] is a newly recognized type of RCC characterized by clear cell morphology and *ELOC*(*TCEB1*) gene mutation. We analyzed one case with a point mutation in *TCEB1* c.218T>A (p.V73E), which is a novel mutation site and has not been reported in *ELOC*(*TCEB1*)-RCC. The case involved a male individual of age 48, whose computed tomography scan of the abdomen indicated the presence of a solid nodule located in the kidney. The tumor cells showed expression of PAX8, CA9, AMACR/P504S, Vimentin, CK7, CD10, FH, INI1(SMARCB1) and ELOC(TCEB1), and ELOC was mainly located in the nucleus. CD117, TFE3, HMB45, and SDHB were not express, and the expression rate of Ki67 was <5%. The novel variant in *ELOC*(*TCEB1*) gene was identified by the next-generation sequencing (NGS) test, subsequently also confirmed by Sanger sequencing. The *ELOC*(*TCEB1*) gene mutation testing is helpful for the diagnosis of this type of RCC. The case further expands our knowledge of the spectrum of *TCEB1* gene mutation in *ELOC*(*TCEB1*)-RCC and enhances the optimization of clinical decision-making.

## 1 Introduction

The molecular features that define clear cell renal cell carcinoma (ccRCC) initiation and progression are being increasingly defined (1). The molecular features that arise from these defects enable categorization of ccRCC into clinically and therapeutically relevant subtypes. The main change in the WHO 2022 classification is the introduction of a new category of molecularly-defined RCC, which includes *TFE3*-rearranged RCC, *TFEB*-rearranged RCC, and *TFEB*-amplified RCC, *FH*-deficient RCC, *SDH*-deficient RCC, *ALK*-rearranged RCC, *ELOC*(*TCEB1*)-mutated RCC, *INI1*(*SMARCB1*)-deficient RCC (2). The transcription elongation factor B (*TCEB1*) gene, encoding the protein elongin C (ELOC), contributes to the Von Hippel-Lindau (VHL) complex to ubiquitinate hypoxia-inducible factor (HIF). Integrated sequencing analysis identified a group of tumors among RCCs characterized by hotspot mutations in *TCEB1* gene S23L, Y79C/S/F/N, I95N or A100P, A106D (3–5). *ELOC*(*TCEB1*)-mutated RCC [*ELOC*(*TCEB1*)-RCC] is a newly recognized type of RCC characterized by clear cell morphology and *TCEB1* gene hotspot mutations, which has been classified as RCC with leiomyomatous stroma (RCCLMS) (1, 6). RCCLMS is a novel subtype of RCC with unique morphologic, immunohistochemical, and molecular characteristics that is distinct from ccRCC and clear cell-papillary RCC. RCCLMS harbors recurrent mutations of *TSC1*/*TSC2*, *MTOR*, and/or *ELOC*(*TCEB1*) genes, consistent with hyperactive MTOR complex; while ccRCC demonstrates primary alterations in *VHL* gene (7). With the increasing application of next generation sequencing (NGS) and other molecular biological detection technologies, it has been possible to determine the oncogenic activation alterations of many solid tumors (8). *ELOC*(*TCEB1*)-RCC is a distinct entity with recurrent hotspot mutations, specific copy number alterations, pathway activation and characteristic morphologic features (2). Here, we find a rare case of *ELOC*(*TCEB1*)-RCC, harboring a novel mutation site in *TCEB1* gene c.218T>A (p.V73E), which can broaden our understanding of RCC genotype and maybe provide treatment options.

## 2 Case presentation

### 2.1 Clinical data

**Figure 1:**
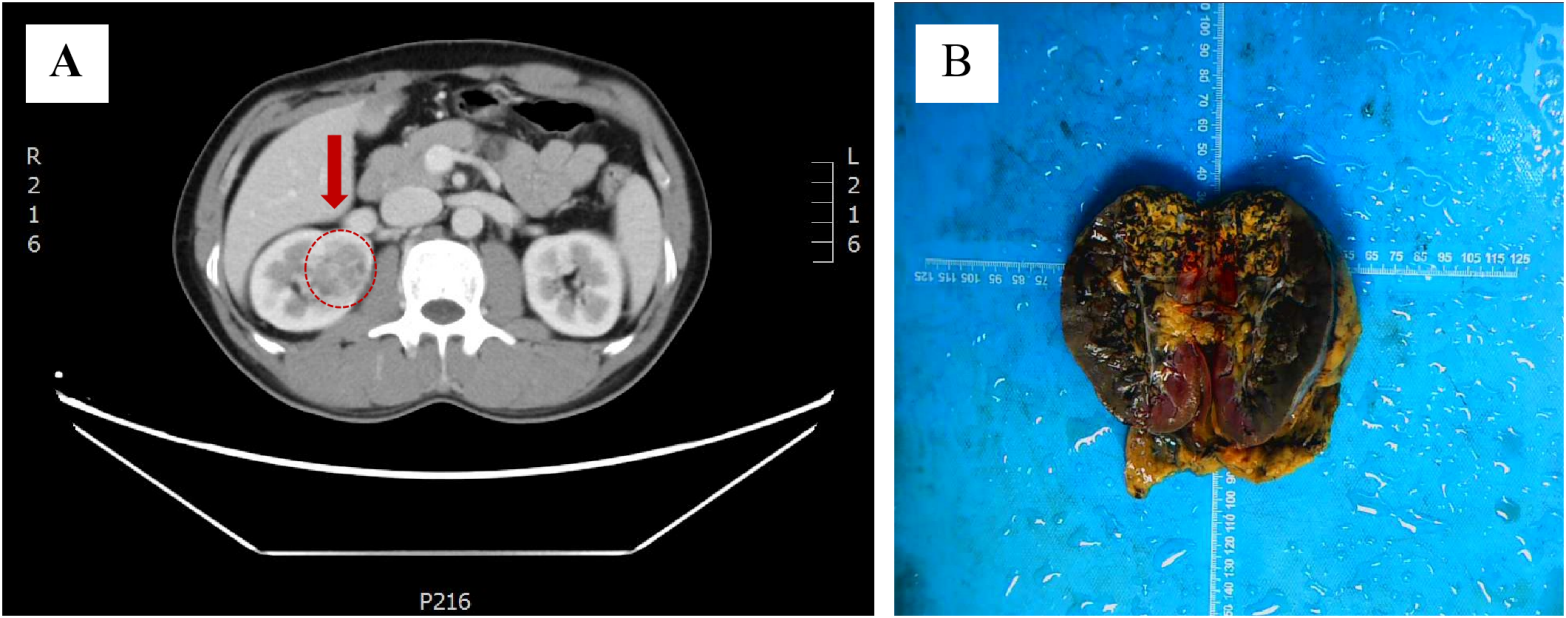
Discovery of *ELOC*(*TCEB1*)-RCC. **(A)** CT scans showed rounded low-density shadows in renal parenchyma. **(B)** Gross observation of *ELOC*(*TCEB1*)-RCC.

A 48-year-old man presented to the urology department with an incidentally discovered renal lesion on screening CT scan (**Figure 1A**), showing rounded nodular low-density shadows in renal parenchyma with a clear boundary, and uneven enhancement was observed on the enhanced CT scan. The TNM stage was observed to be stage I. The patient underwent radical nephrectomy and were followed up for 14 months without any other treatment after surgery. The case has survived till now and showed no evidence of recurrence or metastasis.

### 2.2 Pathological examination

**Figure 2:**
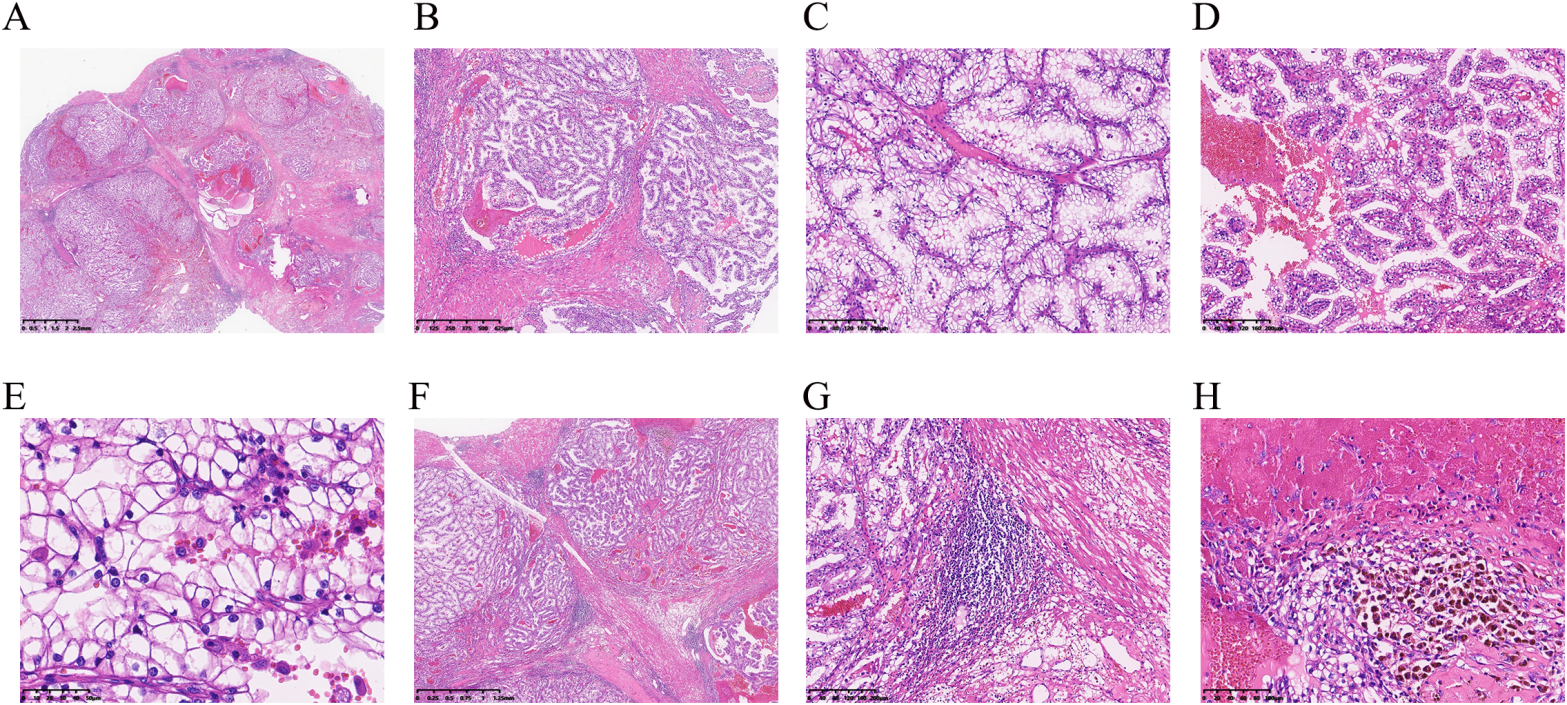
Microscopy of ELOC(*TCEB1*)-RCC. **(A)** Under low-power magnification, the tumor is separated into multinodular masses by thick fibromuscular stroma (×6.6). **(B)** A thick fibrous pseudocapsule rich in smooth muscle can be seen within the tumor (×40). **(C)** Tumor cells show dense arrangement in medium-sized acinar patterns (×100). **(D)** The tumor cells were arranged in short papillary shapes (×100). **(E)** WHO/ISUP nuclear grading for the tumor was grade 1 in the patient, and nuclei were densely stained and irregular in shape (×400). **(F, G)** In this case, focal lymphocyte aggregation (F, ×20) and lymphoid follicle formation (G, ×100) were observed. **(H)** Intratumoral hemorrhage and hemosiderin deposition are observed (×200).

The tumor was located in the renal parenchyma, approximately 3.5 cm in diameter, nodular, gray-yellow or gray-brown, solid in texture and with clear boundaries (**Figure 1B**). All hematoxylin and eosin (H&E) slides from the case of *ELOC*(*TCEB1*)-RCC from Department of Pathology, The Second Xiangya Hospital, were reviewed by two experienced genitourinary pathologists. The tumor of the patient was nodular (**Figure 2A**); a thick fibrous pseudocapsule rich in smooth muscle was visible (**Figure 2B**). The tumor cells were mainly arranged in dense medium-sized acini (**Figure 2C**) or short papillae (**Figure 2D**). The tumor cells had clear boundaries, transparent cytoplasm, irregular or short fusiform nuclei, dense chromatin, unclear nucleoli, slight atypia, and WHO/ISUP nuclear grade 1 (**Figure 2E**). In the tumor of this patient, multifocal lymphocyte aggregation (**Figure 2F, G**) was observed, accompanied by hemorrhage and hemosiderin deposition (**Figure 2H**).

### 2.3 Immunohistochemical staining

**Figure 3:**
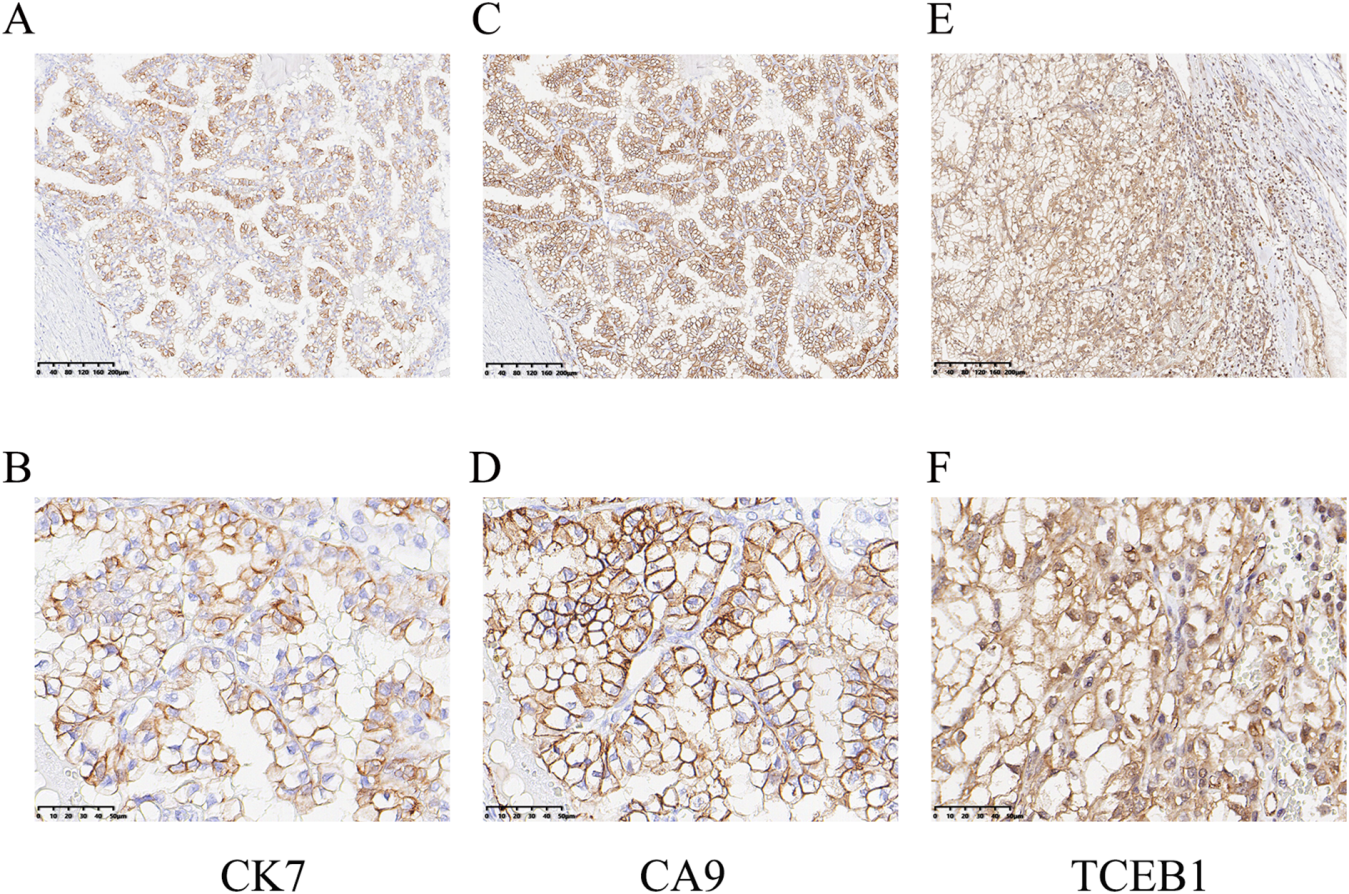
Immunohistochemical staining of ELOC(TCEB1)-RCC. **(A, B)** The tumor cells were positive for CK7 (A, ×100; B, ×400). **(C, D)** Diffuse membrane strong positive for CA9 (C, ×100; D, ×400). **(E, F)** ELOC (TCEB1) was mainly located in the nucleus of tumor cells (E, ×100; F, ×400).

The immunohistochemical stains for carbonic anhydrase-IX (CA9), Paired box 8 (PAX8), Cytokeratin 7 (CK7), common acute lymphocytic leukemia antigen/CD10 (CALLA/CD10) and α-methylacyl CoA racemase/P504S (AMACR/P504S) were performed. The choice of immunohistochemical stains was based on the utility of these markers among certain renal cell carcinomas that may be confused with these *TCEB1*-mutated tumors because of some morphologic overlaps. The immunohistochemical results showed that the tumor was positive for PAX8, CA9 (diffuse box-like positivity), CK7, CD10, P504S, and vimentin. Among them, tumor cells showed moderate positivity for CK7 (**Figure 3A, B**); CA9 showed diffuse strong positive membrane staining, completely outlining the cell membrane (**Figure 3C, D**); ELOC was moderate positive, mainly localized in the nucleus, with varying degrees in staining intensity and range (**Figure 3E, F**). Additionally, in this case, the tumor cells showed Ki67<5%, and did not express CD117, TFE3, HMB45, or SDHB, while expressing FH and INI1(SMARCB1).

### 2.4 *TCEB1* gene mutation detection

**Figure 4:**
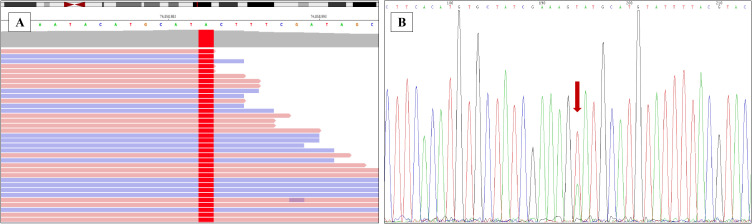
Validating the novel mutation of *ELOC*(*TCEB1*) gene. **(A)** The point mutation of *ELOC*(*TCEB1*) gene in the 73th amino acid was identified by the next-generation sequencing (NGS). **(B)** The point mutation of *TCEB1* gene in the 73th amino acid was verified by Sanger sequencing.

The tumor tissues from lung puncture were made into FFPE samples and used for making pathological sections. Genomic DNA was extracted from FFPE tumor samples using the QIAamp DNA FFPE tissue kit (Qiagen, Hilden, Germany). Mutational hot spots were analyzed using targeted deep sequencing with a capture-based NGS panel obtained from GenePlus Technology Co., Ltd. (Beijing, China). The NGS panel included an assay targeting 1021 genes (*EGFR*/*KRAS*/*ALK* etc.) known to be involved in solid tumors. The 1021 panel covers full exonic coverage for *VHL*, *BAP1*, *PBRM1*, *SETD2*, *FLCN*, *MET*, *PTEN*, *TP53*, *FH*, *SDH*, *ALK*, *TSC1*, *TSC2*, and *MTOR* genes; for *TFE3* gene, it covers partial exons and partial introns; while *TFEB* gene is not covered. Therefore, there may be some limitations in the detection of rearrangement variants of TFE3 and TFEB. DNA sequencing was then performed on the GenePlus Seq-2000 system. The assay can identify various types of genomic alterations, including single base substitutions, insertions/deletions of different lengths, copy number variations, gene fusions, and rearrangements. A point mutation variant in *TCEB1* gene NM_005648.3: c.218T>A (p.Val73Glu) was identified, resulting in a change in the codon 73 from Valine to Glutamic acid (**Figure 4A**). The p.Val73Glu of *TCEB1* gene is a novel mutation site, which has not been reported. In this case, the copy number alterations and loss of heterozygosity of chromosome 8 were not observed.

This novel mutation site was also identified with Sanger Sequencing. *TCEB1* gene was amplified using polymerase chain reaction (PCR). The Primer 5 software was used to design the primers, and the primer sequences are presented below: upstream primer: 5′-TGGATTGCCACCCTAATGAC-3′, downstream primer: 5′-GAATTCAGGAATCTCGGTGGAG-3′. The following PCR reaction conditions were used: pre-denaturation for 1 min at 98°C; (i) denaturation at 98°C for 10 s; (ii) annealing at 55°C for 5 s; (iii) extension at 72°C for 15 s; (iv) repeat (i)–(iii) for 35 times and (v) incubate at 72°C for 10 min. PCR products were identified using agarose gel electrophoresis and then purified. The purified amplification products were sent to Sheng Gong bioengineering (Shanghai, China) co., LTD. After purification, the 3730XL (ABI, Singapore) sequencer was used for sequencing (**Figure 4B**).

## 3 Discussion

*ELOC*(*TCEB1*)-RCC was first reported in 2015, with more than 90% of patients being male (9). Most cases exhibit low-grade malignancy, but 10% of cases show metastasis. This case involves a 48-year-old male, with the tumor located in the right kidney and no metastasis observed (**Figure 1**). *ELOC*(*TCEB1*)-RCC has a broad morphological spectrum, mainly characterized by fibrous smooth muscle tissue separating the tumor into nodular structures (10). The tumor cells have clear cytoplasm and fibrous/fibromuscular stroma (FMS) (or leiomyoma-like stroma), making the tumor appear nodular under low-power microscopy. The architecture is diverse, including solid, acinar, and nested patterns, occasionally with cystic and tubulopapillary structures (11). Importantly, this tumor shows positive CK7 expression with significant variation—expression results differ in some tumors, with as low as 10% to 15% of tumor cells stained, ranging from patchy to diffuse. CA9 (typically with complete membranous staining) and CD10 are consistently immunoreactive. The positivity of CK7 and CA9 is typical and is required for the diagnosis of RCC with FMS (10). These morphological and immunological features are essential criteria for the diagnosis of *ELOC*(*TCEB1*)-RCC. The case is also characterized by fibromuscular tissue dividing the tumor into nodular shapes. Morphologically, it is characterized by branched acinar or tubular structures, with focal scattered short papillary structures (**Figure 2**). Immunologically, tumor cells are positive for CA9 and CK7, and ELOC shows nuclear positivity (**Figure 3**). Additionally, in this case, tumor cells don’t express CD117, TFE3, HMB45, or SDHB, but express FH and INI1(SMARCB1). Thus, we can exclude other molecularly-defined RCC categories, including *TFE3*-rearranged RCC, *FH*-deficient RCC, *SDH*-deficient RCC, and *SMARCB1*-deficient RCC.

*ELOC*(*TCEB1*)-RCC is a subtype of RCC first recognized by the World Health Organization (WHO) in 2022, molecularly characterized by the presence of *TCEB1* gene mutations and the absence of *VHL* gene mutations (12). Most patients with *ELOC*(*TCEB1*)-RCC have a good prognosis, while nuclear pleomorphism and multifocal necrosis may indicate adverse biological behavior (9). The researchers proposed for the first time the significance of immunohistochemical nuclear positive for ELOC and Sanger sequencing in the diagnosis of *ELOC*(*TCEB1*)-RCC *(*3). *TCEB1*-mutated tumors also did not possess any additional recurrent copy number events such as 5q amplifications or 14q or 9p losses which are common in ccRCC, papillary renal cell carcinoma (PRCC), and collecting duct carcinoma (CDC) (3, 13). 5q amplification is one of the most common copy number variations in ccRCC, present in approximately 65%~70% of patients with ccRCC (13). 14q, harboring HIF-1α gene, loss is present in ccRCC (14), and type 1 PRCC with a relatively low incidence (<10%) (15). CDKN2A/2B (9p) deletions are present in ccRCC, type 2 PRCC, and CDC (16). *ELOC*(*TCEB1*)-RCCs are mostly low grade, lack the common chromosomal alterations or gene mutations seen in RCC, including *PBRM1*, *SETD2*, *BAP1*, *TSC1*, *TSC2*, or *mTOR* (11, 17). The *TCEB1* hotspot mutations (Y79C/S/F/N, E92K, A100P, A106D, and C112Vfs∗3) were all located within or close to the VHL-binding domains in ELOC protein (9, 18). In this case, a novel mutation site (p.V73E) in *TCEB1* gene was identified in the tumor by NGS and Sanger Sequencing (figure 4). Researchers have reported the crystal structure of VHL bound to a Cul2 N-terminal domain and ELOC protein (residues 17-112), which is the minimal domain required for VHL binding (18). The mutation site of *TCEB1* gene V73E is exactly located within the VHL-binding domain. We believe that the novel variant of *TCEB1* c.218T>A (p.V73E) is a driving mutation and harbors oncogenic potential, which is one of the potential pathogeneses in *ELOC*(*TCEB1*)-RCC. However, it remains to be further validated.

*ELOC*(*TCEB1*)-RCC exhibits an indolent biological behavior with limited metastatic potential. If no clinical aggressiveness, surgical resection is usually curative. For tumors in stages T1-T2, adjuvant therapy is not required after surgery, while adjuvant treatment regimens for advanced-stage tumors are the same as those for non-ccRCC (12). Currently, no record of the novel mutation site c.218T>A (p.V73E) in *TCEB1* gene has been found in public population databases (e.g., 1000 Genomes, GO ESP, Gnomad) or in COSMIC, a database of human cancer driver genes. This mutation scores 0.9987 in AlphaMissense and is predicted to be a Strong Pathogenic mutation. The codon altered by this mutation site is located in the VHL-binding domain of ELOC, which belongs to a relatively conserved region of the protein. The functional prediction software SIFT indicates that this mutation is deleterious. Overall, the case was diagnosed as *ELOC*(*TCEB1*)-RCC through morphological features, immunophenotypic characteristics, and molecular pathological analyses. The patient has been free of recurrence or metastasis for more than a dozen months after undergoing radical nephrectomy.

In conclusion, we report a very rare case of *ELOC*(*TCEB1*)-RCC in an adult with a novel variation in *TCEB1*: c.218T>A (p.V73E). The presence of FMS, and immunohistochemical positive for PAX8, CK7, CA9, CD10, and AMACR/P504S, and positive ELOC nuclear staining may be an important indication for the diagnosis of this disease. The detection of *TCEB1* gene mutation is helpful for the diagnosis of *ELOC*(*TCEB1*)-RCC through Sanger sequencing or next generation sequencing.

## Data Availability

Datasets are available on request. The raw data supporting the conclusions of this article will be made available by the authors, without undue reservation.
